# Assessment of Flooring Renovations on African Elephant (*Loxodonta africana*) Behavior and Glucocorticoid Response

**DOI:** 10.1371/journal.pone.0141009

**Published:** 2015-11-04

**Authors:** Sarah A. Boyle, Beth Roberts, Brittany M. Pope, Margaret R. Blake, Stephen E. Leavelle, Jennifer J. Marshall, Andrew Smith, Amanda Hadicke, Josephine F. Falcone, Katrina Knott, Andrew J. Kouba

**Affiliations:** 1 Department of Biology, Rhodes College, Memphis, Tennessee, United States of America; 2 Conservation and Research Department, Memphis Zoological Society, Memphis, Tennessee, United States of America; University of Houston, UNITED STATES

## Abstract

Captive African (*Loxodonta africana*) and Asian (*Elephas maximus*) elephants can experience foot pathologies and arthritis. As a preventative measure against these pathologies and to alleviate the potential discomfort due to concrete substrates, some zoological institutions have renovated elephant housing to increase the amount of natural or shock-absorbent substrates. The objective of this study was to compare behavioral (diurnal and nocturnal) and glucorticoid (e.g., serum cortisol) responses of three female African elephants before, during, and after renovation to their indoor housing floor to assess whether renovations had short-term effects on the elephants’ behavior and stress physiology. Behavioral data were collected using scan-sampling methods, and activity budgets were constructed for each of the three elephants. In addition, the duration of all lying rest activities were recorded. Weekly serum cortisol concentrations were determined with enzyme immunoassay (EIA). Overall, eating was the most prevalent behavior exhibited outdoors during the day, while resting (either in a lying or standing position) were most common during the indoor, nocturnal periods. Although variation existed among the three elephants, all three females spent significantly more time walking and less time eating during the day after the completion of the renovations. The extent to which the three elephants exhibited nocturnal lying rest behavior varied among the elephants, with the oldest elephant exhibiting the least amount (an average of 13.2 ± 2.8% of the nightly behavioral scans) compared to the two younger elephants (an average of 34.5 ± 2.1% and 56.6 ± 2.8% of the nightly behavioral scans). There was a significant increase in lying rest behavior for one elephant and standing rest for a second elephant following renovations. Baseline cortisol concentrations prior to renovations were 3.0 ± 0.4 ng/ml, 4.5 ± 0.5 ng/ml, and 4.9 ± 0.5 ng/ml for the three elephants. Cortisol concentrations remained baseline for two of the elephants throughout and after the renovation period, while one elephant that was pregnant had elevated cortisol during construction. Cortisol concentrations for the pregnant elephant remained higher than baseline once she was introduced to the new flooring and allowed back into the building, but these values were closer to the cortisol concentrations before renovations than during construction. Our findings demonstrate that individual elephants can vary in their behavioral and physiological responses to exhibit modifications. Given that the elephants walked more during the day, two of the three elephants had an increase in rest behavior during the night, and there were minimal changes in cortisol response after the flooring renovations, we conclude that the flooring renovations overall had a positive impact on animal welfare.

## Introduction

Male African (*Loxodonta africana*) elephants weigh an average of 6,000 kg and females 5,000 kg, while Asian (*Elephas maximus*) elephants average 4,500 kg and 3,500 kg for males and females, respectively [1]. As a result of this weight and pressure, elephants can experience problems with foot health and arthritis [1–3]. In a 2006 survey of 78 Association of Zoo and Aquarium (AZA) facilities, approximately one-third of the institutions reported having at least one case of elephant foot pathology annually [2]. Furthermore, 13% of elephants (18% of Asian elephants and 8% of African elephants) in the AZA survey were diagnosed with arthritis, with this disease being significantly more common in older elephants [2]. To decrease the prevalence of these foot pathologies and joint problems, elephant facilities have focused on regular foot and nail care, increased exercise plans, and improved flooring substrates [2–4].

The daily distance that African elephants walk in the wild varies from 3–38 km at a rate of 0.2–1.1 km/h [5]. Comparable with some of the distances and rates walked by elephants in the wild, studies of the daily movement distances of captive elephants have reported ranges from 3–11 km with rates of 0.3–0.6 km/h [5–7]. In captivity, a strong negative correlation between the amount of exercise that captive elephants receive and the prevalence of foot pathology has been noted [2]. However, the substrates on which the elephants walk in captivity can vary greatly from the natural substrates in the wild. As of 2006, 69% of indoor areas for elephants in AZA facilities were concrete, 24% rubber, 5% sand, and 2% other, while outdoor areas were 47% dirt, 37% sand, 8% concrete, 5% rock, 1% rubber, and 2% other [2]. Recently some zoological institutions have changed elephant flooring from concrete to substrates that mimic natural materials, or to substrates that are durable, soft, and more shock-absorbent [3,4,8]. When provided with natural substrates in captivity, elephants can benefit from improved foot and joint health due to increased blood flow, filing of the nails and foot pad, and increased movement of foot muscles, tendons and joints [9]. After the change from concrete to rubberized flooring of the indoor enclosure at the Oregon Zoo, Asian elephants exhibited increased locomotion behavior, and the elephants decreased the proportion of time spent in a recumbent (lying rest) position [8]. To date there are limited published studies evaluating the impacts of flooring changes on African elephant behavior or physiology. Therefore, the health benefit of rubber flooring on the overall captive population of elephants is largely unknown.

Glucocorticoid assays are commonly performed to assess stress in wild and captive animals [10–12]. In the wild, variations in elephant glucocorticoid concentrations can change depending on the type of stressor, season, group size and composition, and the individual’s sex, age, and social dominance rank, but these patterns are not universal [10,13–16]. Studies of captive African and Asian elephants have shown that glucocorticoid concentrations can differ among individuals [17–20], and that glucocorticoid fluctuations can occur depending on the time of day and type of stressor [18,19,21,22]. Although the current study is the first to our knowledge to measure glucocorticoid concentrations in elephants in relationship to exhibit renovations, previous studies have examined the relationship between exhibit design and glucocorticoid concentrations in other species. Wielebnowski et al. [23] found that the fecal glucocorticoid concentrations of clouded leopards (*Neofelis nebulosa*) were negatively correlated with enclosure height and the number of hours that keepers spent with the animals, and fecal glucocorticoid concentrations were positively related to when the animals were held on public display or housed near predators. In studies examining glucocorticoid concentrations in the context of construction, responses have varied in felids (black leopard, *Panthera pardus*; serval, *Leptailurus serval*; Afghan leopard, *Panthera pardus saxicolor*; and snow leopard, *Uncia uncial*; [24]) and in giant pandas (*Ailuropoda melanoleuca*; [25]). Furthermore, the relationship between glucocorticoid concentrations and behavioral patterns (e.g., agonistic behavior, stereotypic behavior) have not been consistent across studies of captive animals [19,20,24].

The aim of this study was to document diurnal and nocturnal behavior, in combination with blood glucocorticoid (i.e. serum cortisol) concentrations, in African elephants before, during, and after the installation of new rubberized flooring in their indoor housing. This approach allowed us to examine active and inactive behaviors across 24-h periods, which is important because elephant activity budgets often differ between the day and night [26,27]. In the wild, African elephants often spend most of the day hours in active behaviors such as eating [28,29], while sleeping (lying down) behavior occurs primarily at night [29]. Furthermore, the collection of behavioral and glucocorticoid data are important measures for assessing elephant welfare [26,30,31]. Although the main goal of the flooring renovation that replaced concrete with a shock-absorbent rubber substrate was to minimize foot and joint problems in the long term, our study examined the short-term effects of the renovations on the elephants to determine 1) if the renovations resulted in changes in the elephants’ behaviors, and 2) if the glucocorticoid concentrations varied with such changes in behavior and flooring modification. We hypothesized that flooring renovations positively impact elephant behavior and glucocorticoid concentrations. We predicted that after renovations the elephants would exhibit an increase in resting behaviors (i.e., lying rest, standing rest) during the night in their indoor exhibit, and an increase in active behaviors (i.e. eating, exploratory, walking) during the day in their outdoor exhibit. We also predicted that glucocorticoid concentrations would be lowest after renovations, suggesting a reduced stress response. The examination of behavioral and physiological measures can indicate to what extent the implementation of shock-absorbent flooring can provide immediate benefit to joint health and animal welfare through limiting the time that elephants apply weight and pressure to joints. Initial positive responses to flooring renovation hold promise for the long-term health benefits that minimize foot pathology and discomforts of arthritis in aging captive elephants.

## Materials and Methods

### Study subjects and housing

**Fig 1:**
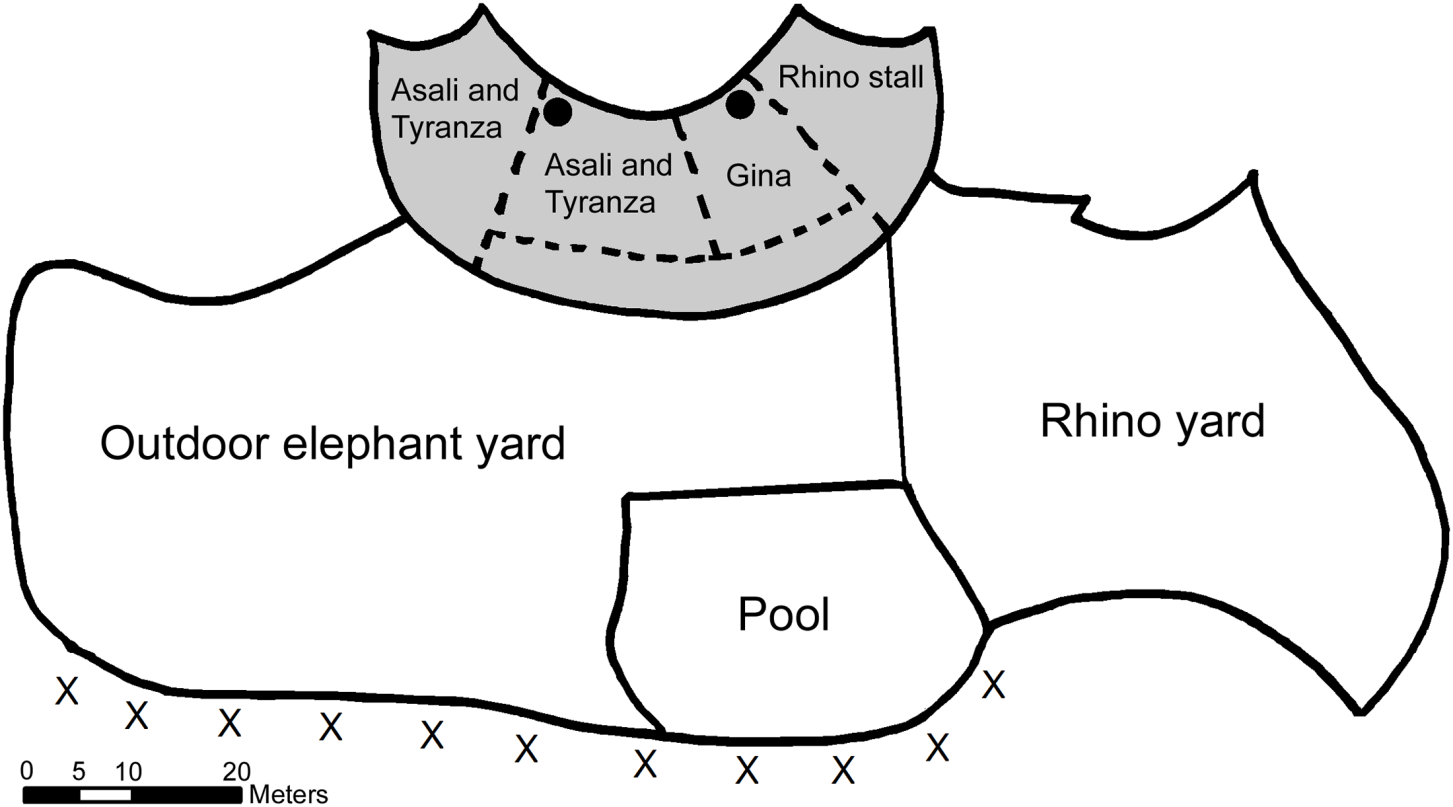
Map of the indoor and outdoor exhibits. The indoor area where flooring was replaced is noted in grey. Circles represent position of indoor video cameras for recording nocturnal behavior. Observations of diurnal outdoor behavior were taken in locations noted by X along the perimeter of the exhibit.

Three female African elephants reside at the Memphis Zoological Society in Memphis, Tennessee: Tyranza (wild-born in 1965; 46 years old at time of the study), Gina (wild-born in 1983; 28 years old at time of the study), and Asali (captive-born in 1985; 26 years old at time of the study). During the study period, the outdoor exhibit (2,200 m^2^) primarily consisted of a compact sand substrate. The exhibit also included a concrete pool and round, concrete structures for shade. The outdoor exhibit was split so that the elephants could have access to one side of the yard, or full access to both yards. During the study period the elephants had access to three stalls and a shift area in the indoor exhibit. When elephants were inside for the night, Tyranza and Asali had access to two stalls and the shift area, while Gina was housed alone in an adjoining stall that allowed for visual interaction and tactile contact over the stall wall (Fig 1). In November 2011, the Memphis Zoo replaced the old concrete floor with soft-composite resilient flooring (Specialty Coating Solutions, Matthews, NC). The upper layer of the new flooring was a tough, high-density elastomer that was chemically bonded to the light-weight, low-density elastomer of the lower layer. In addition to the flooring changes, new stall shift doors replaced the old ones, allowing for more open visual and tactile communication.

Data collection consisted of live observation of diurnal outdoor behaviors, video observation of nocturnal indoor behaviors, and weekly serum collection for cortisol assays.

### Diurnal outdoor behavioral data collection

Diurnal outdoor behavioral data were collected before (October 1 –November 6, 2011), during (November 8 –November 15, 2011), and after (November 17 –December 11, 2011) renovations, for an average of 3 h per day, with 71 h of diurnal data collected for each of the three elephants. Data collection spanned a period from 09:00 to 16:00 h with equal representation of morning, mid-day, and afternoon hours so that data represented all times of day and all activity patterns of the elephants.

Scan sampling [32] methods at two-min intervals were used to record the behavior of each elephant in sight. The original ethogram consisted of 21 behaviors, which were condensed into six behavioral categories (lying rest: lateral recumbence; standing rest: upright and stationary; eating: manipulation and consumption of food; walking: movement forward or backward of more than half a body length; exploratory: use of trunk to touch surrounding feature, excluding conspecifics and self; other; all other behaviors, including agonistic, social, and stereotypic). These six behavioral categories were based on the ethogram used by Brockett et al. [33], with the exception of ‘exploratory’ behavior, which we added for comparison to Meller et al. [8]. Activity budgets for each elephant were calculated based on the percent of behavioral scans associated with each of the six behavioral categories. These activity budgets were calculated for the entire data set as well as for the three separate time periods (before, during, and after renovations).

Although changes in flooring, changes in the stall doors, and the disruption of daily activities due to the construction itself could all play roles in potential changes in the elephants’ behaviors, our focus centered on analyzing behaviors that would be most associated with the flooring changes (nocturnal lying rest and standing rest before and after renovations). Analysis also included other behaviors (diurnal active behaviors before, during and after renovations) that may have been related to flooring changes, given that if there was increased resting activity at night that the elephants may have exhibited more active behavior during the day in their larger outdoor exhibit. Some of these behaviors could have been related to changes in the elephants’ schedules due to construction and may not be directly attributable to the flooring changes.

### Nocturnal indoor behavioral data collection

Two mounted video cameras recorded continuous footage of Gina, Asali and Tyranza while in their indoor stalls. A gate separated the two stalls, but the elephants could reach over the gate into the next stall. Although Asali and Tyranza also had access to a second stall and a shift area, difficulties with nighttime visibility of those additional video cameras restricted the amount of data collected when elephants were in these locations.

Video data were downloaded every 15 min from 17:30 to 08:00 h for 13 evenings before (9/30/2011–11/5/2011) and 13 evenings after (11/16/2011–12/3/2011) the flooring renovations for a total of 377 h for 26 nights. During renovations the elephants did not have access to the indoor area, so there were no data for the period of time when the elephants were outside during the night. We set the size of each data download to 4 MB, and downloads averaged a duration of 5.5 min. Every 15 min the behavior of each elephant in view was recorded using the interval scan sampling method (see Diurnal outdoor behavioral data collection), following the same ethogram as with the outdoor data collection. Activity budgets were calculated for each of the three elephants overall, as well as before and after renovations. Because behavioral data for Asali and Tyranza were obtained in only one of their two stalls due to difficulties with video clarity, a t-test was conducted to determine whether or not there was a difference in the amount of time that each animal spent in the visible stall, as such a difference could impact our conclusions if one of the two animals spent more time out-of-view of the video camera. Overall Asali and Tyranza were present for an average of 66.9% and 63.6% of the video behavioral scans, respectively, and there was no difference between the time that the animals were in view (*t* = 1.23, *df* = 50, *P* = 0.23). Asali and Tyranza were visible in 63.1 ± 3.3% and 56.5 ± 3.0% of the behavioral scans, respectively, before the flooring renovation occurred and 70.7 ± 2.8% and 70.7 ± 2.7%, respectively, after the renovation. Therefore, we felt confident of our ability to evenly compare behavioral results based on the percentages of video scans when the two elephants were in view of the working video camera.

Further analysis of the behavioral video data included tallying the number of lying rest ‘events’ that occurred each night, with one ‘event’ defined as all consecutive behavioral scans of lying rest behavior. Lastly, the duration of each lying rest event was quantified, as well as the time from the start of the first lying rest event to the end of the last lying rest event.

### Serum collection, extraction, and enzyme immunoassays

Serum samples from the three African elephants were collected weekly from the vein on the caudal aspect of the ear. Serum was used in this study because all animals were well-conditioned to the blood collection as part of the normal management routine for monitoring serum progesterone throughout the estrous cycle. Collection typically occurred between 09:00 to 10:00 h, 1 h after the morning feeding and prior to routine bathing. Blood was collected in 10 ml serum separator tubes with a 20 g needle and a vacutainer. Samples were allowed to clot at room temperature for 1 h before centrifuging the samples (1500 g) for 10 min. Serum was then transferred into cryovials and stored frozen (-70°C) until extracted and assayed for cortisol. Serum samples were collected for 16 wk prior to renovations to 11 wk after the new flooring was installed. Given that the elephants were locked indoors at night during video collections for the behavioral study, and that being locked inside could impact glucocorticoid concentrations, more weeks of serum samples prior to renovations than after renovations were analyzed.

Serum was extracted using diethyl ether. Briefly, 200 μl of serum was mixed with 800 μl of diethyl ether (5:1 v/v), vortexed for 2 min, and allowed to settle for 5 min before snap freezing for 10 min at -70°C. The liquid solvent solution was then quickly poured into a clean tube. The remaining pellet was thawed and re-extracted with another 800 μl of diethyl ether added to the original tube and frozen, and the solvent poured off. The two extracted portions were combined and placed in a vacucentrifuge to speed dry for 45 min. Dried samples were resuspended at room temperature with 800 μl of phosphate buffer (phosphate-buffered saline, 0.1% BSA; pH 7.0) at a 1:5 dilution.

The concentrations of the immunoreactive cortisol metabolites in serum extracts were determined using a single antibody competitive enzyme immunoassay (EIA). The EIA utilized cortisol standards (hydrocortisone; Sigma-Aldrich, St. Louis, MO), a polyclonal cortisol antibody (R-4866), and a cortisol horseradish peroxidase (HRP) label (antibody and HRP were obtained from C. Munro, University of California Davis) previously validated for African elephant urine [34]. Cortisol EIA methods and cross reactivities were described by Young et al. [35]. Briefly, polystyrene 96-well microtiter plates (NUNC, Thermo Scientific, Rochester, NY) were coated with 50 μl cortisol antibody diluted 1:8500 in coating solution (0.05 M NaHCO_3_, pH 9.6) and stored overnight at 4°C. Nine cortisol standards were prepared by a 1:2 serial dilution from the stock (1000–3.90 pg/well) and serum extracts were further diluted in phosphate buffer prior to assay (1:25 total). Standards, serum extracts, and two controls were run in 50 μl per well triplicates. Cortisol HRP (1:80,000) was immediately applied to compete for binding sites on the antibodies, and the plates were allowed to equilibrate at room temperature for 1 h. Plates were washed 5 times with wash buffer and developed with 100μl of peroxidase substrate azino-bis-3-ethylbenxthiazoline-6-sulfonic acid (ABTS, 40 mM) and 1.6 mM hydrogen peroxide in 0.05M citric acid (pH 4.0) for colorimetric detection of HRP binding activity. Plates were incubated and shaken for 10 to 15 min while covered at room temperature and then optical density was read with an MRX Revelation microplate reader (ThermoScientific, Rochester, NY) using the 405 nm filter. The concentrations of the serum hormone metabolites were determined by the inverse of the bound fraction as compared to a standard curve. Hormone metabolite concentrations in serum were reported as ng/ml.

The assay was validated for the serum extraction by demonstrating that the serial dilution of serum extract pools were parallel to the standard curve. Recovery of standard hormone concentrations (500, 250, 125, 62.5, 31.25, 15.6, 7.8, 3.9 pg/well) added to pooled serum extracts was 109%; y = 1.044x-1.7849, r^2^ = 0.998. Inter- and intra-assay coefficients of variation for the assay were determined by the addition of a low (~80% total binding) and high (~25% total binding) concentration control ran on each plate. The inter-assay coefficient of variation was 7.94% (low) and 2.92% (high), and intra-assay variation was below 10%.

### Analyses

Data were analyzed using SPSS 22.0, and results were presented as mean ± standard error (SE) and *P* ≤ 0.05 considered significant. Analyses of overall trends were conducted using nonparametric statistical tests, but due to the small sample size (*n* = 3) and because behavioral patterns can vary significantly between individual elephants [27], analyses were also conducted for each elephant separately. Because multiple measures of the same individual violate the assumption of independence that is required for most statistical analyses [36], patterns within each individual elephant were examined using 95% confidence intervals (CI) so that measures falling outside of the 95% CI for the baseline data (data from before renovations began) were considered different from baseline measures [25]. Although results from individual patterns should not be used to make broad generalizations about all captive elephants, the findings are important for a preliminary understanding of the elephants’ behavioral and physiological responses to changes in their environment.

For each of the six behavioral categories (lying rest, standing rest, eating, walking, exploratory, and other), the percentage of behavioral scans representing each behavior was calculated for each of the three elephants. For the outdoor, diurnal behavior, a Friedman Test was used to examine differences in values before, during, and after renovations for each of the six behavioral categories. Patterns for individual elephants were then assessed using the 95% CI from the ‘before’ data. For the indoor, nocturnal behavior, a Wilcoxon Signed Ranks Test was used to compare the six behavioral categories before and after renovations. A Wilcoxon Signed Ranks Test was also used to test for differences in the mean number of ‘lying rest’ events per night, the mean duration of ‘lying rest’ events, and the time from the first to the last ‘lying rest’ event before and after renovations. Patterns for individual elephants were similarly assessed using the 95% CI from the ‘before’ data.

Baseline values for cortisol were established using a reiterative process so that values greater than two standard deviations from the mean were excluded and considered peaks [37]. To test for differences before, during, and after renovations, a Friedman Test was used. Patterns for individual elephants were then assessed using the 95% CI from the ‘before’ data.

The Memphis Zoo and Rhodes College do not require an IACUC for remote animal observations that do not impact the regular routine of captive wildlife in their care; nor, does the Memphis Zoo require an IACUC for weekly blood draws on their three females cows as this is part of their routine veterinary health care and maintenance procedures for monitoring estrous cycle and/or pregnancies.

## Results

### Outdoor diurnal behavior

**Fig 2:**
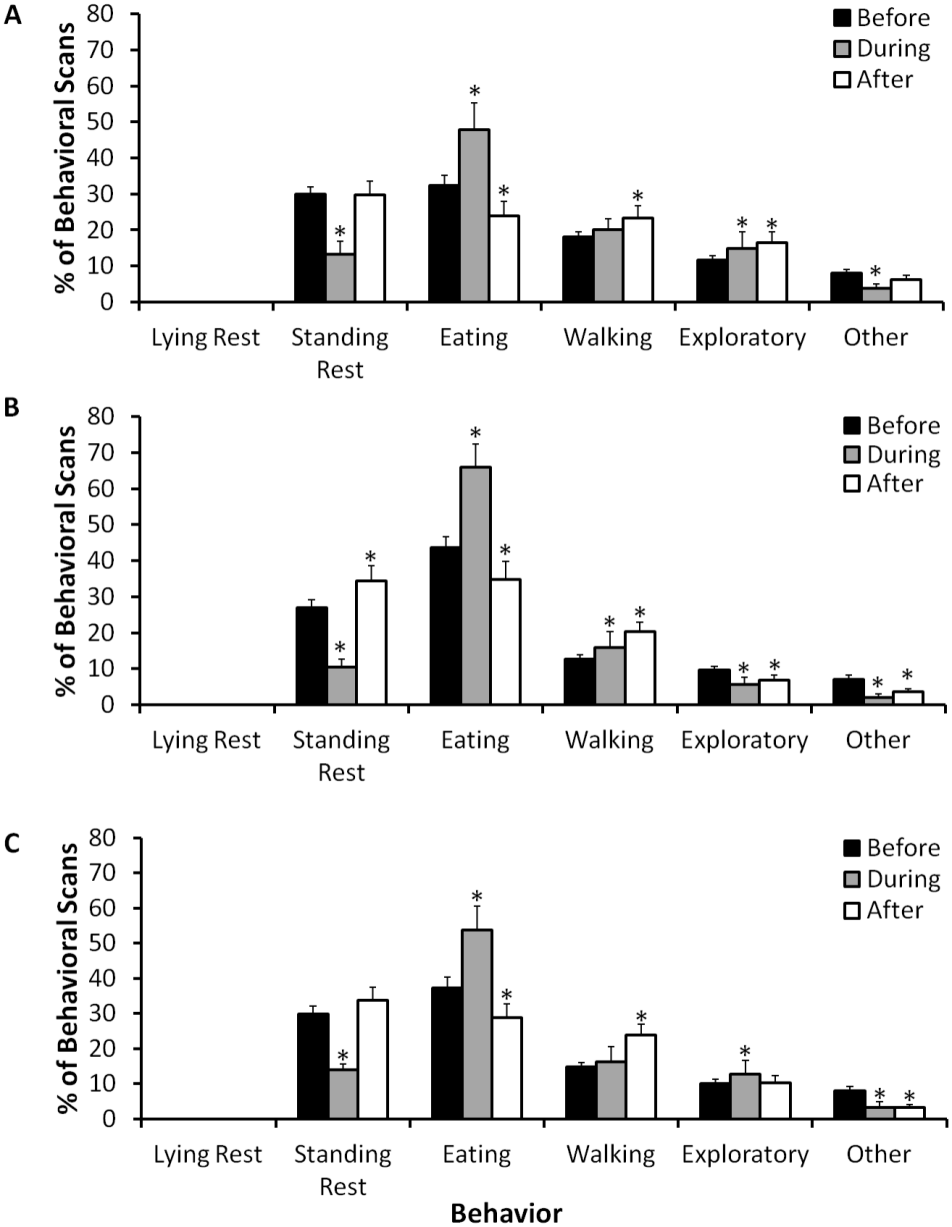
Diurnal outdoor activity budgets (mean ± SE) of three female African elephants (A: Asali; B: Gina; C: Tyranza) before, during, and after installation of rubberized flooring in their indoor facilities. Outdoor behavioral data collection occurred from approximately 09:00 to 16:00 h. An asterisk (*) indicates that the mean value is outside the 95% confidence interval (CI) for the data before renovations.

Overall, eating was consistently the most common behavior exhibited outdoors during the day (Fig 2). In total across the entire study period, all three elephants spent most of their time eating, followed by standing rest, walking, exploratory, and then other (Table 1). Social behaviors, as well as agonistic behaviors, comprised less than 1% of the behavioral scans for all three elephants, and none of the elephants exhibited lying rest during the outdoor, diurnal behavioral scans.

**Table 1:** Percent of diurnal, outdoor behavioral scans (mean ± standard error) the elephants spent engaged in each behavioral category throughout the entire duration of the study.

Elephant	Lying Rest	Standing Rest	Eating	Walking	Exploratory	Other
**Asali**	0.0	27.9 ± 1.8	31.8 ± 2.3	19.8 ± 1.3	13.4 ± 1.2	7.0 ± 0.8
**Gina**	0.0	27.1 ± 1.9	43.8 ± 2.6	15.2 ± 1.2	8.3 ± 0.8	5.5 ± 0.8
**Tyranza**	0.0	29.1 ± 1.8	36.8 ± 2.3	17.5 ± 1.3	10.4 ± 1.1	6.2 ± 0.7

When comparing overall behavioral patterns in the three elephants before, during and after renovations, the percent of behavioral scans spent eating (*Χ* ^2^ = 6.00, *n* = 3, *df* = 2, *P* = 0.05) and walking (*Χ* ^2^ = 6.00, *n* = 3, *df* = 2, *P* = 0.05) differed across the three time periods. For eating behavior, values were greatest for all three elephants during renovations, followed by before renovations and then after renovations (Fig 2). For walking behavior, values increased from before to during and then to after renovations. There were no overall patterns in standing rest (*Χ* ^2^ = 4.67, *n* = 3, *df* = 2, *P* = 0.097), exploratory (*Χ* ^2^ = 0.67, *n* = 3, *df* = 2, *P* = 0.72), or other (*Χ* ^2^ = 4.67, *n* = 3, *df* = 2, *P* = 0.097) behaviors. Examining each elephant’s behavioral patterns individually, during the renovations Asali’s standing rest decreased, eating increased, exploratory increased, and other decreased when compared to her behaviors before the renovations began. Walking did not differ between before and during renovations. After the renovations, Asali’s eating decreased, walking increased, and exploratory increased when compared to the before values (Fig 2A). All of Gina’s behaviors during renovations also differed from the before renovation values. After renovations, Gina’s standing rest increased, eating decreased, walking increased, exploratory decreased, and other decreased in comparison with the before values (Fig 2B). For Tyranza, all behaviors except for walking differed during renovations when compared to the before values. After renovations, Tyranza’s eating decreased, walking increased and other decreased when compared to the before renovations values.

### Indoor nocturnal behavior

All three elephants engaged in resting (lying and/or standing) behaviors more often (range: 61.9%–75.8% of the behavioral scans) than any other behaviors during the indoor nocturnal periods (Table 2). Asali and Gina both spent the greatest amount of time in lying rest behavior, followed by standing rest, exploratory, and then eating. Walking and other behaviors each represented 5% or less of the behavioral scans for both elephants. Tyranza spent most of the time exhibiting standing rest behavior, followed by exploratory, lying rest, and eating. Similar to the other two elephants, Tyranza spent less than 5% of the time engaged in walking or other behaviors.

**Table 2:** Percent of nocturnal, indoor behavioral scans (mean ± standard error) the elephants spent engaged in each behavioral category throughout the entire duration of the study.

Elephant	Lying Rest	Standing Rest	Eating	Walking	Exploratory	Other
**Asali**	34.5 ± 2.1	27.3 ± 1.8	14.4 ± 1.8	2.4 ± 0.5	20.3 ± 1.8	1.0 ± 0.3
**Gina**	56.6 ± 2.8	19.2 ± 1.8	7.8 ± 0.9	1.9 ± 0.4	9.4 ± 1.1	5.1 ± 1.2
**Tyranza**	13.2 ± 2.8	57.0 ± 3.1	10.5 ± 1.4	2.3 ± 0.4	16.6 ± 2.0	0.4 ± 0.2

Two agonistic encounters occurred during the nocturnal behavioral scans, and both encounters occurred between Asali and Gina above the gate separating the two stalls. Only one scan of stereotypic behavior was documented during the behavioral scans and it involved Tyranza pacing repetitively while tossing her trunk. Social behavior consisted of 0.7 ± 0.2% of the nocturnal behavioral scans for Asali, 1.1 ± 0.4% of the scans for Gina, and 0.2 ± 0.1% of the scans for Tyranza.

**Fig 3:**
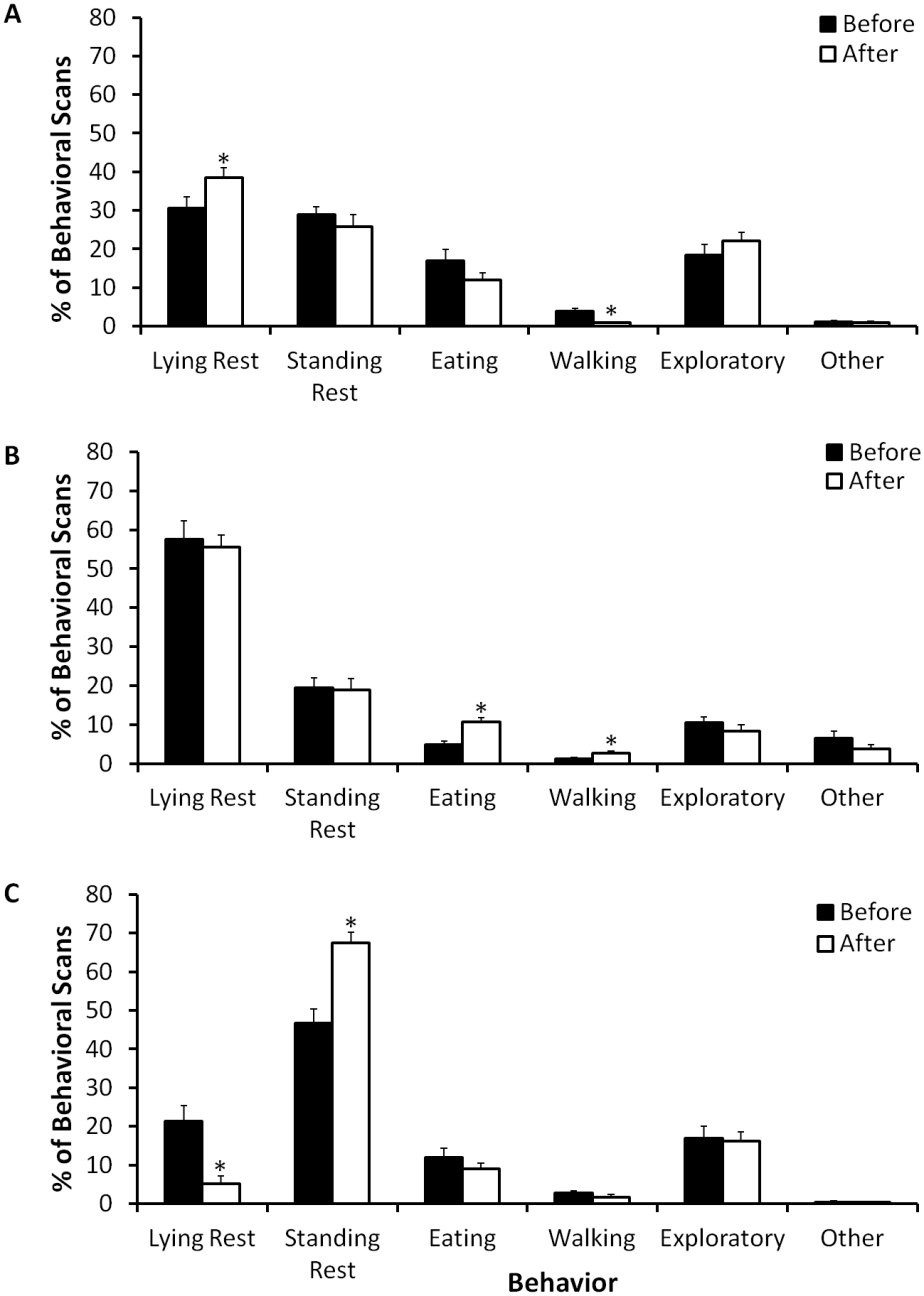
Nocturnal indoor activity budgets (mean ± SE) of three female African elephants (A: Asali; B: Gina; C: Tyranza) before and after installation of rubberized flooring in their indoor facilities. Indoor behavioral data collection occurred from approximately 17:30 to 8:00 h, with data collection ending when husbandry by an animal keeper began. During renovations the elephants were not allowed indoors. An asterisk (*) indicates that the mean for after renovations is outside the 95% CI for the data before renovations.

Examined together, the three elephants did not differ before and after renovations in their nocturnal lying rest (*Z* = -0.54, *n* = 3, *P* = 0.59), standing rest (*Z* = 0.00, *n* = 3, *P* = 1.0), eating (*Z* = 0.00, *n* = 3, *P* = 1.0), walking (*Z* = -0.54, *n* = 3, *P* = 0.59), exploratory (*Z* = 0.00, *n* = 3, *P* = 1.0), or other (*Z* = -1.60, *n* = 3, *P* = 0.11) behaviors. Examining each elephant individually, Asali exhibited an increase in lying rest and a decrease in walking behaviors after renovations (Fig 3A). There were no differences before and after renovations in standing rest, eating, exploratory, or other behavioral categories. After renovations Gina exhibited an increase in eating and walking, yet there were no differences in lying rest, standing rest, exploratory, or other behavioral categories (Fig 3B). After renovations Tyranza exhibited a strong decrease in lying rest and an increase in standing rest, but there were no differences in eating, walking, exploratory, and other behavior (Fig 3C).

**Fig 4:**
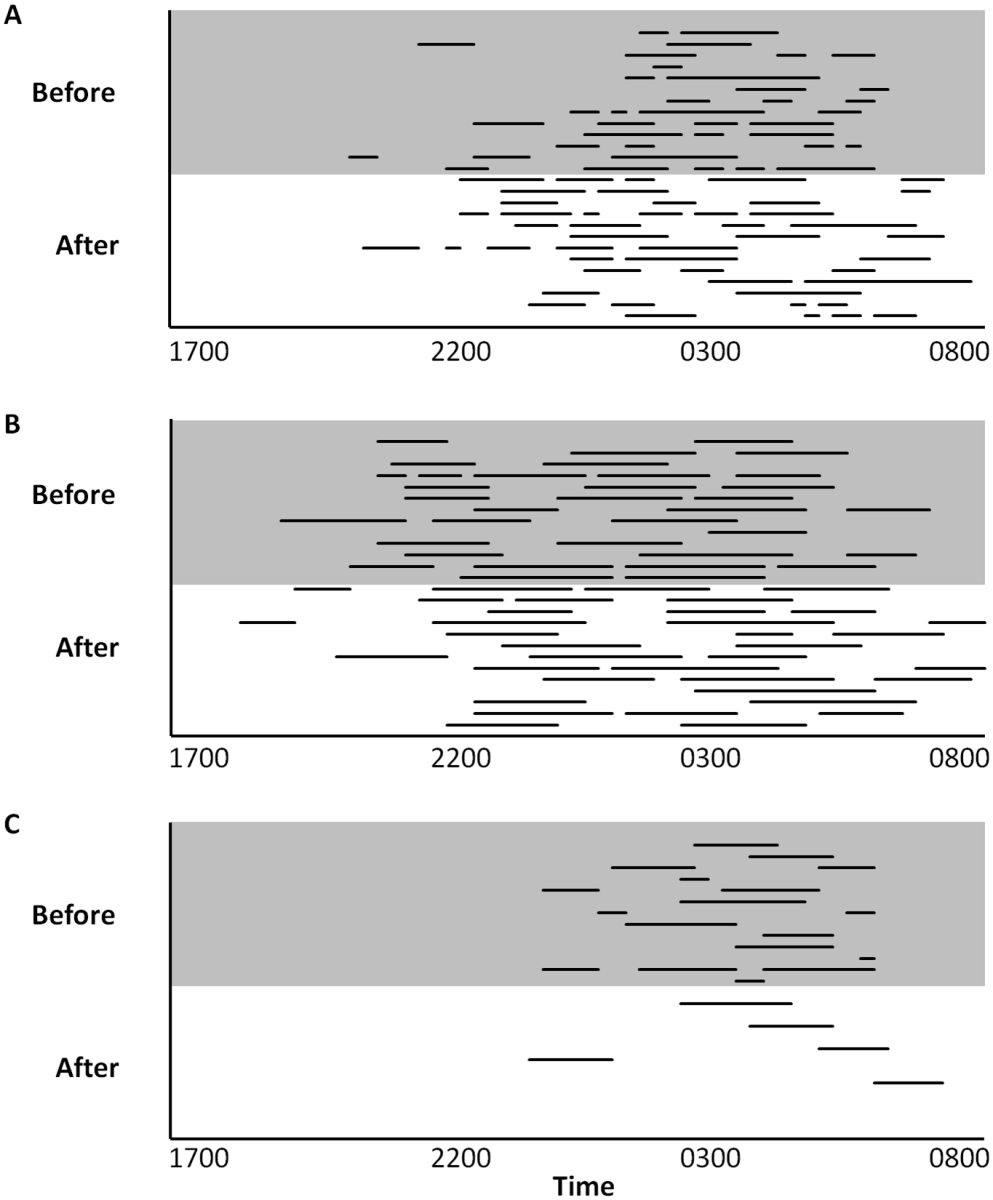
Frequency, duration, and time of day of lying rest (recumbent) behavior 13 days before (shaded) and after (not shaded) installation of the new flooring. Horizontal lines represent when across the 26 days the behavior was exhibited by (A) Asali, (B) Gina, and (C) Tyranza.

Asali and Gina exhibited lying rest behavior in 100% of the nights sampled, but Tyranza exhibited this behavior for only 69% of the nights sampled (100% of the nights before renovations and 38% of the nights after renovations). The time of night when lying rest behavior was exhibited was more restricted for Tyranza than for Gina, who exhibited the behavior starting in early evening, and Asali, who exhibited the behavior starting in late evening (Fig 4).

Throughout the entire study period, Asali exhibited 3.3 ± 0.2 lying rest events per night that lasted on average 64.2 ± 4.6 min. Asali’s duration from the first lying rest event to the last event was 318.8 ± 21.5 min. Gina exhibited 2.7 ± 0.2 lying rest events per night that lasted on average 122.5 ± 4.5 min. Gina’s duration from the first lying rest event to the last event was 431.0 ± 27.9 min. Tyranza exhibited 0.9 ± 0.2 lying rest events per night that lasted on average 79.0 ± 7.9 min. Tyranza’s duration from the first lying rest event to the last event was 124.4 ± 25.0 min.

**Fig 5:**
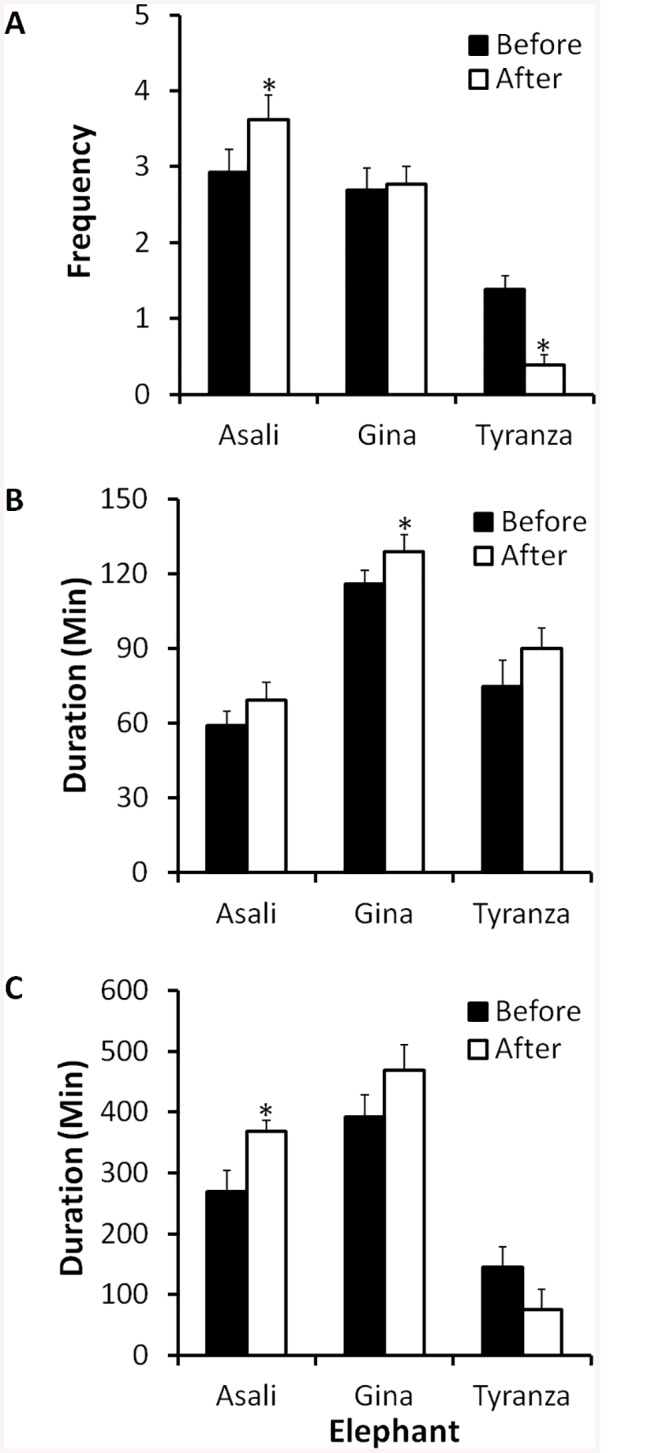
Mean ± SE of (A) frequency of sleeping (‘lying rest’) events per night; (B) duration of events; and (C) duration between the first and last events each night before and after renovations. An asterisk (*) indicates that the mean for after renovations is outside the 95% CI for the data before renovations.

Examining the three elephants together, there were no overall differences before and after renovations in the mean number of lying rest events per night (*Z* = 0.00, *n* = 3, *P* = 1.0) or the duration from the first to the last event (*Z* = -1.07, *n* = 3, *P* = 0.29). The mean duration of each lying rest event was also not significantly different before and after renovations (*Z* = -1.60, *n* = 3, *P* = 0.11), but when the three elephants were examined individually, Gina did show a significant increase in the duration of lying rest events, and Asali and Tyranza approached a similar trend (Fig 5). Asali exhibited more lying rest events/night and had a greater duration from first to last event after the renovations than before the renovations. Gina increased the mean duration of lying rest events after the renovations, and Tyranza decreased the number of lying rest events/night after the renovations (Fig 5). The overall pattern was an increase in lying rest behavior for Asali and Gina after renovations, with an overall decrease in the occurrence of lying rest behavior by Tyranza. However, the duration of the lying rest events for Tyranza was greater than these events for Asali, but Asali had a much greater frequency of such events (Fig 5).

### Cortisol

Baseline values for cortisol varied from 3.0 ± 0.4 ng/ml for Tyranza, to 4.5 ± 0.5 for Asali, to 4.9 ± 0.5 for Gina (Table 3). When examining overall patterns with all three elephants, there were no differences in cortisol before, during, and after renovations (Friedman: *Χ* ^2^ = 2.67, *n* = 3, *df* = 2, *P* = 0.26). Analysis of samples from only before and after renovations, also demonstrated no statistical difference between the two time periods (*Z* = -1.60, *n* = 3, *P* = 0.11). When examining each elephant individually, neither Asali nor Tyranza had cortisol values during and after renovations that exceeded the 95% CI for the baseline values. However, Gina’s cortisol values during and after renovations were greater than the baseline values, but her values after renovations were 65% of the values during renovations (Table 3).

**Table 3:** Mean ± standard error (SE) of serum cortisol (ng/ml) across periods of renovation in the elephant house.

Elephant	Baseline: Before	95% CI for Baseline	During^a^	After
**Asali**	4.5 ± 0.5	3.5–5.6	3.9	4.9 ± 0.7
**Gina**	4.9 ± 0.5	3.8–6.1	12.4*	8.1 ± 0.7*
**Tyranza**	3.0 ± 0.4	2.2–3.8	3.4	3.7 ± 0.4

^a^Only one serum collection occurred during renovations.

*Value is outside of the 95% confidence interval (CI) for the baseline value.

**Fig 6:**
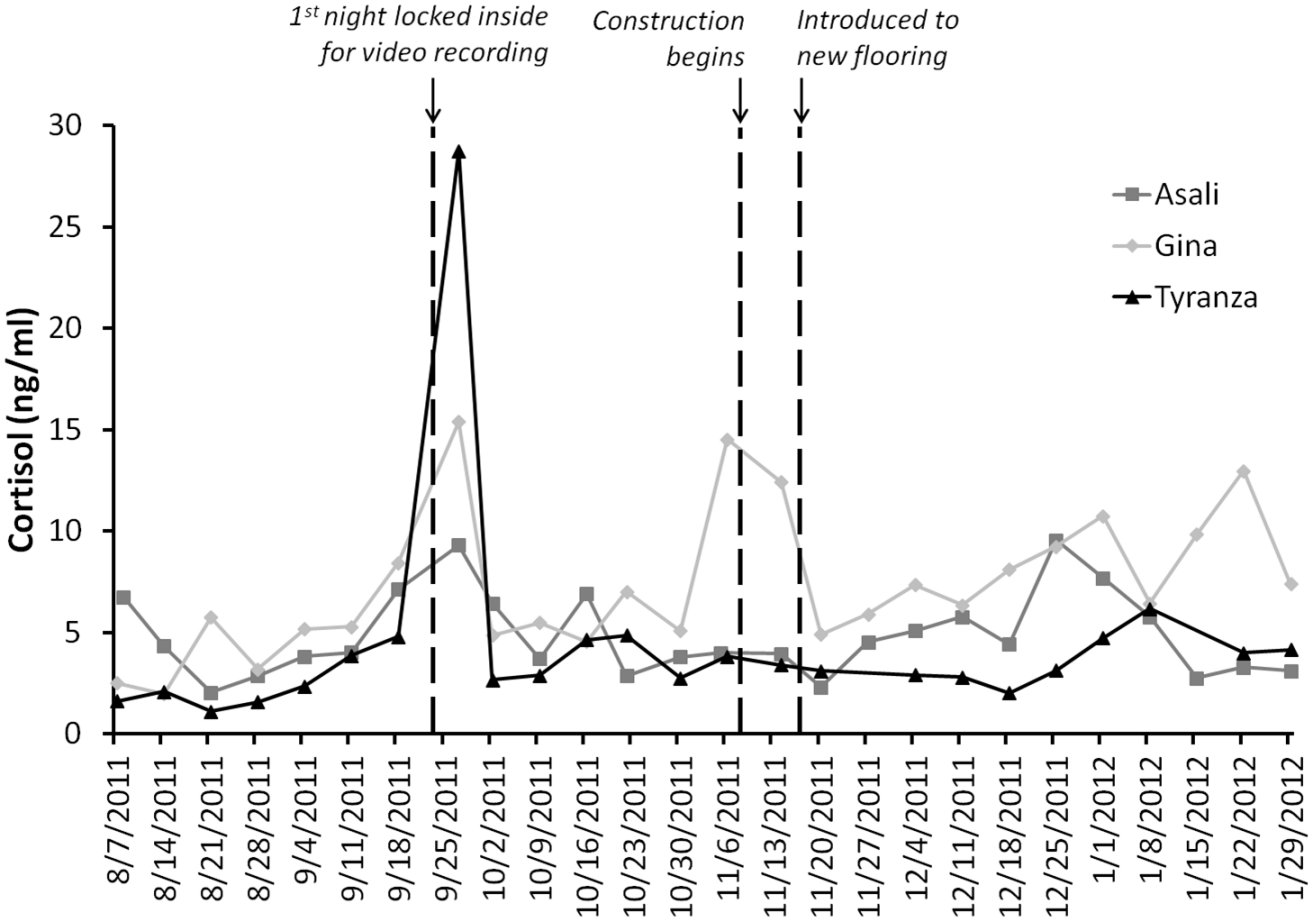
Cortisol (ng/ml) profile for three female African elephants. Profiles represent before (8/7/2011 to 11/7/2011), during (11/8/2011 to 11/15/2011), and after (11/16/2011 to 1/29/2012) renovation to the elephant house.

Peaks in cortisol, as determined by being greater than 2 SD from the mean, occurred for all three animals on 09/27/2011 (Fig 6), which was shortly after they were first locked inside overnight for video recording. Tyranza did not exhibit any other cortisol peaks during the study. Asali had one additional peak after renovations and exposure to the new floor (12/25/2011). Gina had six additional peaks, one immediately before renovations began (11/6/2011), one during renovations (11/14/2011), and four after renovations (12/25/2011, 1/1/2012, 1/15/2012 and 1/22/2012).

## Discussion

These results show that the three African elephants at the Memphis Zoo varied in behavioral and physiological measures before, during, and after the flooring renovations meant to improve the health care of the animals. The general patterns for the three individuals included (1) an increase in diurnal eating during renovations, followed by a decrease after renovations; (2) an increase in diurnal walking after renovations; and (3) an increase in the frequency of lying rest events after renovations for two of the three elephants. Nocturnal behavioral differences also existed among the three elephants, with Asali exhibiting more lying rest behavior after the flooring renovations, Tyranza exhibiting less lying rest behavior but more standing rest behavior, and Gina staying consistent in her resting behavior before and after renovations. Furthermore, cortisol concentrations remained similar before, during, and after renovations for Asali and Tyranza, but cortisol concentrations increased during and after renovations for Gina. Previous studies have also found variations among individual elephants in behavior [27] and cortisol [17–20].

### Flooring changes and behavior

Diurnal and nocturnal behaviors differed in prevalence, with eating being the most common behavior during the day and resting (either lying rest or standing rest) being the most common nocturnal behavior. Horback et al. [26] and Posta et al. [27] also found eating to be the most common diurnal behavior in captive elephants, and in wild elephants eating is often the most common behavior [28,29]. However, Horback et al. [26] found eating to be the most common nocturnal behavior and the percentage of behavioral scans engaged in lying or standing rest was lower in both Horback et al. [26] and Posta et al. [27] than compared to the current study. Such differences between diurnal and nocturnal activity budgets in these studies highlight the importance that future studies measure both diurnal and nocturnal behavior, when possible, in order to gain a full picture of the animals’ responses to changes in their environment. Our finding of an increase in diurnal eating during renovations may not be a direct response to the renovations, but rather to changes in husbandry schedules as the elephants received all of their food outside during the renovation period when the elephants could not access the indoor exhibit.

We found an increase in lying rest or standing rest for two of the three elephants, while Meller et al. [8] found an increase in standing rest and a decrease in lying rest in some locations. Variations in resting behaviors among elephants may be due to multiple factors including animal age, hierarchy, and preference of resting positions. Gina exhibited the greatest percentage of behavioral scans in lying rest behavior, with the greatest mean duration of lying rest events and the greatest duration from first to last events. Although there was not a difference before and after renovations in the percentage of behavioral scans that Gina spent in a lying rest position, the duration of her lying rest events and the duration from the first to the last event increased or showed a trend of increasing after renovations. It would be expected that the activity budget would reflect the increases in lying rest duration, but the lack of such differences may be due to the limited number of behavioral scans that were visible each night. It is unknown whether Gina’s pregnancy during the course of the study was a contributing factor to her greater prevalence in resting.

The duration of lying resting events by Asali were on average half the duration of Gina’s events, but these resting periods occurred more frequently. This resting pattern may indicate that Asali was getting up and down more often throughout the night. After the flooring renovations Asali spent a greater percentage of time in a lying rest position and for a greater duration throughout the night, suggesting that the rubber flooring had a positive effect on the amount of rest she obtained at night. Social interactions leading to restlessness may have contributed to the different resting pattern for Asali as she was the youngest of the elephants and also less dominant.

Captive African elephants have been documented living into their mid-50s [38], and Tyranza was 46 years old at the time of the study, 18 years older than the second-oldest elephant, Gina. Tyranza spent less time in lying rest than the other two elephants, and was the only individual to decrease lying rest behavior after the renovations. In a study of four elephants ranging in age from 4 to 33 years old, McKnight [39] found that the 33-year-old elephant spent less time lying down, and got up and down from a lying position less often than the younger elephants. In a study of a 27-year-old female and her 3-year-old son, Posta et al. [27] documented that the son exhibited nocturnal lying down behavior for 42% of the behavioral scans while the mother did so for only 26% of the scans. Schwammer et al. [40] also documented less lying down behavior in older individuals, and some individuals were never documented lying down. Therefore, Tyranza’s lower baseline lying down values before renovations began could have been due to her advanced age. The increase in standing rest behavior after the flooring renovations may have been due to the new flooring that allowed Tyranza to rest more comfortably in a standing position rather than getting up and down from a recumbent position on the floor. It is noteworthy, however, that after flooring renovations that even Tyranza exhibited a trend towards longer lying rest durations although she was less likely to engage in lying behavior than the other elephants. Given that arthritis is more common in older than younger elephants [3] and Tyranza is an older elephant, the shock-absorbent new flooring substrate will hopefully minimize the pressure on her joints as she ages. Because all three elephants in this study were healthy at the time of study and did not exhibit any foot pathologies, the flooring renovations were preventative measures for improving foot health. Long-term monitoring of the foot and joint health of these elephants, as well as elephants in other facilities, could provide a better understanding of the long-term effects of flooring renovations on elephant health.

The period of peak lying down behavior through the night (2300 h to 0500 h) was similar to the pattern of lying down behavior through the night documented by Horback et al. [26], Tobler [41], and Wilson et al. [42] for elephants in captivity, and by Wyatt and Eltringham [29] for elephants in the wild. However, the period of sleeping behavior began earlier and ended later in the current study than in these other three studies. There was no indication by Horback et al. [26], Tobler [41], and Wilson et al. [42] that the elephants were housed overnight in buildings with rubber floors, so the differences between the prevalence of lying rest behavior in the current study compared to the three other studies could potentially be due to the flooring substrate, or other factors (e.g., time of year, husbandry practices) that vary between institutions. We found the duration from the first to the last lying rest event increased after renovations for Asali and showed a strong trend of increasing with Gina (mean value for after renovations exceeded the 94.7% CI), further suggesting that the rubberized flooring may extend the duration of lying rest behavior throughout the night.

The increase in diurnal walking after flooring renovations was comparable to findings by Meller et al. [8], that six Asian elephants increased their diurnal locomotion in some areas of the indoor housing after installation of new rubberized flooring. Furthermore, nocturnal walking did not increase in either Meller et al. [8] or the current study. Such an increase in diurnal walking from the current study and that of Meller et al. [8] may indicate that the animals had more energy during the day due to increased resting during the night. Neither Meller et al. [8] nor the current study observed diurnal lying rest behavior. This lack of diurnal lying rest behavior in relation to flooring improvements should not be seen as an exclusion of this behavior from daytime activities. In a study of two captive African elephants, Posta et al. [27] documented them lying down during the day indoors, but not outdoors, and Adams and Berg [43] documented captive elephants lying down outside during the day.

Lack of data from the third stall and shift area may have impacted our results for nocturnal behavior for Asali and Tyranza, given that the elephants could have fed or rested in the other stall. However, Gina exhibited a similar pattern of not exhibiting as much eating behavior during the night, and most of Gina’s behavior was captured on one video camera. Furthermore, there was a clear decrease in Tyranza’s lying rest behavior after renovations, and there were no differences between the time Asali and Tyranza spent on video. Therefore, even with these limitations, the data for behaviors within the visible stalls appear to give an overall snapshot of the elephants’ activity budgets and behavioral patterns.

### Flooring changes and cortisol

Average cortisol concentrations for Asali, Gina, and Tyranza (Table 3) were all within the range (2.50–12.51 ng/ml) of serum cortisol concentrations for female African elephants reported by Meyer et al. [44]. Tyranza had the lowest cortisol concentrations throughout the study, followed by Asali and then Gina. In comparing cortisol concentrations before, during, and after renovations, neither Tyranza nor Asali exhibited differences between their baseline values and the values during and after renovations and less than 8% of their total cortisol samples exceeded baseline values. The greatest concentration of cortisol for Tyranza and Asali occurred on a day (9/25/2011) soon after the elephants were first locked inside for the nocturnal video recording. Thereafter, cortisol concentrations for both elephants returned to baseline. Given that the cortisol concentrations for Asali and Tyranza did not differ from baseline during or after the renovations, suggests that the changes in flooring substrate, including the construction itself, resulted in minimal stress to these elephants.

Gina had the highest cortisol concentrations, and 27% of Gina’s samples exceeded baseline values. There are many possible reasons why Gina had greater cortisol concentrations than the other two elephants, and that her cortisol concentrations increased as the study progressed. For example, personality differences between elephants has been related to differences in glucocorticoid response [18]. Gina was involved in more agonistic encounters than the other two elephants, although the overall the percentage of behavioral scans classified as agonistic was very low (< 0.5% of all scans). Gina was also pregnant during the study, which could have impacted her cortisol concentrations [44]. Other elephant studies, however, reported no correlation between cortisol concentrations and an individual’s agonistic behavior [19], stereotypical behavior [20], or pregnancy status [45]. Regardless of the causal factors for greater cortisol concentrations during and after renovations, the maximum peak cortisol concentrations for Gina during and after the renovations were less than the maximum peak values before the renovation, suggesting that the renovations were unlikely to adversely affect her health. Glucocorticoid concentrations in this study, therefore, can serve as baseline data for African elephants in ongoing and future studies of elephant endocrinology.

## Conclusions

Flooring renovations appeared to have a positive impact on the behavior of captive African elephants, and a neutral impact on their glucocorticoid response. Rubberized flooring may help increase walking behavior during the day, thereby allowing for increased blood circulation to the feet, and improved wearing of the foot pads and nails. Such shock-absorbent flooring may also encourage lying rest behavior and result in an increased duration of resting bouts. For aging elephants that tend to minimize the amount of time in a lying rest position, rubberized substrates are anticipated to be more comfortable for joints and foot pads while in standing rest positions. This study provides an initial assessment of how flooring renovations can improve the welfare of animals in captivity. Further examinations that combine long-term behavioral, glucocorticoid, and physiological (e.g. scoring foot and joint health) monitoring of elephants from different age classes and social groupings, and across multiple institutions, are needed to provide further guidance for direct and indirect improvements to animal health and welfare.
